# Proportion of cancer cases and deaths attributable to potentially modifiable risk factors in Peru

**DOI:** 10.1186/s12885-024-12219-4

**Published:** 2024-04-15

**Authors:** Jhony A. De La Cruz-Vargas, Willy Ramos, Willer Chanduví, Lucy E. Correa-López, Nadia Guerrero, Joan Loayza-Castro, Irene Tami-Maury, Diego Venegas

**Affiliations:** 1 Instituto de Investigaciones en Ciencias Biomédicas (INICIB), Universidad Ricardo Palma, Lima, Perú; 2 The University of Texas Health Science Center at Houston, Houston, USA; 3 Facultad de Ciencias e Ingeniería, Universidad Peruana Cayetano Heredia, Lima, Perú

**Keywords:** Cancer, Population attributable fraction, Modifiable risk factors, Lifestyle, Preventive medicine, Peru

## Abstract

**Background:**

Limited evidence exists on the population attributable fraction (PAF) of cancer cases and deaths in Latin America. In Peru several studies have been published regarding the PAF of various risk factors and their associated diseases. The objective of this study was to estimate the fraction of cancer cases and deaths attributable to potentially modifiable risk factors in Peru in 2018, before the COVID-19 pandemic in the population of 15 years old and older.

**Methods:**

An ecological study was conducted using the prevalence of exposure of the Peruvian population to modifiable risk factors for cancer, the relative risk associated with each factor, and the number of cancer cases and deaths in 2018 as inputs. We used the Parkin formula with a Montecarlo statistical simulation model to calculate the PAF and confidence intervals. The number of new cancer cases and deaths attributed to each risk factor was determined by multiplying the number of cases and deaths in each gender by the PAF of each risk factor.

**Findings:**

In Peru, 38.5% of new cases (34.5% in men and 42% in women) and 43.4% of cancer-related deaths (43.4% in men and 43.4% in women) were attributable to modifiable risk factors. The number of cancers attributable was 25,308 (10,439 in men and 14,869 in women) and the number of deaths attributable to cancer was 14,839 (6,953 in men and 7,886 in women). The predominant modifiable risk factors contributing to the highest number of cases and deaths were HPV infection (4,563 cases, 2,409 deaths), current tobacco use (3,348 cases, 2,180 deaths), and *helicobacter pylori* infection (2,677 cases, 1,873 deaths). Among the risk factors, oncogenic infections constituted the group with the highest PAF (16.6% for cases, 19.2% for deaths) followed by other unhealthy lifestyle factors (14.2% for cases, 16.7% for deaths), tobacco (7.2% for cases, 7.2% for deaths) and ultraviolet radiation (0.5% for cases, 0.3% for deaths).

**Conclusions:**

Prior to the COVID-19 pandemic, 38.5% of cancer cases and 43.4% of cancer-related deaths in Peru were linked to modifiable risk factors in the population of 15 years old and older. Most preventable cancer cases and deaths were related to oncogenic infections, primarily caused by HPV and *helicobacter pylori*, followed by tobacco and obesity.

## Introduction

Cancer constitutes a significant public health challenge in Peru due to the uncontrolled high prevalence of risk factors and disparities in accessing oncological services. This leads to delayed diagnoses and unequal treatment, ultimately increasing the risk of premature deaths among Peruvians [1–4]. In recent decades, transitions and social determinants in demographics, epidemiology, commercial diets, and nutrition, have significantly shaped Peru’s health profile, resulting in the dominance of non-communicable diseases such as cancer [5]. In addition, socio-economic factors such as poverty, education, gender, urbanization/rurality, ethnicity, race, environmental factors, and other health determinants influence Peruvians’ exposure to risk factors and access to healthcare [6]. Despite this, the country’s morbidity, and mortality continue to be affected by communicable diseases [1, 5–7].

According to the estimates from the International Agency for Research on Cancer (IARC) published by the Global Cancer Observatory, Peru registered 66,669 new cancer cases and 34,570 cancer-related deaths in 2018 (prior to the COVID-19 pandemic) [2, 3].

Potentially modifiable risk factors significantly contribute to numerous cancers, and the current estimation of this proportion within a population, known as the PAF, constitutes a valuable tool for prioritizing cancer prevention and control programs and interventions [8] and, in turn, allows us to estimate the percentage of cases that could have been avoided if exposure to associated risk factors had been minimized compared to the reference level [9]. There has been PAF studies assessing modifiable cancer risk factors in various regions such as North America (United States and Canada) [10, 11], Europe (England) [12], Asia (China, Japan) [13, 14] and the Middle East [15]. These studies have reported PAFs ranging from 23 to 45% for cancer cases and from 41 to 51% for cancer-related deaths. However, most cancer studies are focused on PAF for cancer cases, with only a few considering PAF for cancer-related deaths [10, 13, 14, 16].

0n the contrary, there are very little evidence of PAF for cases and deaths for cancer in Latin-American countries and do not exits PAF’s studies in Peru that consider all the risk factors and allow the estimation of the total number of cancer cases and deaths attributable to said risk factors. However, do exits PAF studies of some risk factors and derivate diseases (as can be observe on the tobacco consumption [17, 18] mortality tendencies for different cancer’s type, as gastric [19], kidney [20], cervical cancer [21], mesothelioma [22]). The sole Latin-American’s country that have one study of PAF for cancer is Brazil [16]; on the other hand, Chile has one PAF study that considers only lifestyle risk factors, not including oncogenic infections [23].

The objective of this study is to estimate the fraction of cancer cases and deaths in Peru attributable to potentially modifiable risk factors in 2018, prior to the COVID-19 pandemic.

## Methods

An ecological study was conducted using data on the Peruvian population’s prevalence of exposure to modifiable risk factors for cancer, alongside cancer incidence and mortality preceding the COVID-19 pandemic in the population of 15 years old and older.

The modifiable risk factors reported in this study (Table 1), were obtained from a systematic review of the global burden of cancer attributable to risk factors 2010–2019 [24], in addition to primary published PAF studies [12–17, 23].

### Prevalence of modifiable risk factors, cancer cases, and deaths

The prevalence of the Peruvian population’s exposure to modifiable risk factors for cancer was obtained from the following data sources:

- Population surveys: The Demographic and Family Health Survey (ENDES 2018) provided data on cigarette and alcohol use, fruit and vegetable consumption, overweight and obesity among individuals aged 15 and above.
- Research articles: These articles yielded information on the prevalence of oncogenic infections, sedentary behavior, and other relevant factors.
- University thesis repositories: In some cases, specific information from both undergraduate and postgraduate thesis was obtained, mainly focusing on rare risk factors or those with limited studies in the Peruvian population, such as the prevalence of red meat and processed meat consumption (However, there are no thesis citations within the article).

### Relative risk (RR)

The relative risk associated with each potentially modifiable risk factor was determined with a systematic search across databases including PUBMED, SCOPUS, EMBASE, COCHRANE, and SCIELO, prioritizing metanalysis, cohort and case-control studies, favoring recent studies covering American countries and controlling for cofounding variables through a multivariate statistical analysis. In cases where the systematic search did not yield studies with RR data for the potentially modifiable risk factors, research presenting odds ratios (OR) was used as a statistical approximation to RR.

### Estimate of the PAF

The calculation of the PAF was performed using the formula described by Parkin et al. [25]:

$$ \frac{{({p_1} \times {\rm{ER}}{{\rm{R}}_1}) + ({p_2} \times {\rm{ER}}{{\rm{R}}_2}) + ({p_3} \times {\rm{ER}}{{\rm{R}}_3}) \ldots + ({p_n} \times {\rm{ER}}{{\rm{R}}_n})}}{{1 + [({p_1} \times {\rm{ER}}{{\rm{R}}_1}) + ({p_2} \times {\rm{ER}}{{\rm{R}}_2}) + ({p_3} \times {\rm{ER}}{{\rm{R}}_3}) \ldots + ({p_n} \times {\rm{ER}}{{\rm{R}}_n})]}} $$

*p*_1_ represents the proportion of the population at exposure level 1 and subsequent levels, while ERR_1_ is the excess relative risk (relative risk − 1) at exposure level 1 and subsequent levels. The researchers calculated the PAF for the absence or decrease of risk factors. ERR was determined as the natural logarithm of the reciprocal of the relative risk, expressed by the formula:

$$ ERR=\text{l}\text{n}\left(\frac{1}{RR}\right)$$

Cancer cases and deaths data in Peru were obtained from the Global Cancer Observatory (GLOBOCAN-Cancer today) which publishes the IARC estimates based on population cancer registry data from the year 2018 [3].

The calculation of the number of cancer cases and deaths attributed to each risk factor, categorized by gender, involved multiplying the number of cancer cases and deaths per gender by the PAF.

**Table 1:** Potentially modifiable risk factors considered for each type of cancer

TOPOGRAPHY	MODIFIABLE RISK FACTORS
Mouth, pharynx (C00-C14)	HPV infection, tobacco use, alcohol use, low fruit and vegetable consumption
Esophagus (C15)	Current tobacco use, obesity, sedentary behavior, alcohol use
Stomach (C16)	*Helicobacter pylori i*nfection, current tobacco use, obesity, red meat consumption, processed meat consumption
Colorectal (C18-C21)	Current tobacco use, sedentary behavior, alcohol use, overweight, obesity, red meat consumption, processed meat consumption, low fruit and vegetable consumption.
Liver (C22)	Hepatitis B virus, Hepatitis C virus, current tobacco use, obesity, overweight
Gallbladder (C23)	Obesity, overweight
Pancreas (C25)	Tobacco use, alcohol use, obesity
Larynx (C32)	HPV infection, current tobacco use, low fruit and vegetable consumption
Lung (C34)	Current tobacco use, passive exposure to tobacco smoke, sedentary behavior, low fruit and vegetable consumption
Melanoma of the skin (C43)	Ultraviolet radiation
Kaposi Sarcoma (C46)	HHV-8 infection, HIV
Breast (C50)	Tobacco use, passive exposure to tobacco smoke, obesity, overweight, sedentary behavior
Cervix (C53)	HPV infection
Endometrium (C54.1)	Obesity, overweight, sedentary behavior
Ovaries (C56)	Obesity, overweight
Penis (C60)	HPV infection
Kidney (C64)	Tobacco use, overweight, obesity
Bladder (C67)	Current tobacco use, overweight, obesity
Hodgkin’s Lymphoma (C81)	Epstein Barr virus, HIV, current tobacco use
Non-Hodgkin’s Lymphoma (C82-C85, C96.3)	Epstein Barr virus, HIV, Hepatitis C virus
Leukemias (C91-C95)	Tobacco use, obesity, overweight

In case of cervical cancer and Kaposi sarcoma, a PAF of 100% was assumed, directly associated with VPH and VIH infections [26]. For skin melanoma, the PAF estimated by the IARC for the Peruvian population was derived from their “Cancers attributable to UV radiation” statistics available on the World Cancer Observatory website [27].

From an ethical point of view, this study did not imply risks as it utilized aggregate prevalence data from population surveys, relative risks from metanalysis and scientific journal articles, and the IARC cancer incidence and mortality estimates for Peru. Therefore, it did not require an informed consent. Additionally, the study received approval from the Research Ethics Committee from the Medical School of Ricardo Palma University (Expedited Review: PI-019-2023).

## Results

In 2018, before to the COVID-19 pandemic, approximately 38.5% of new cancer cases in Peru were attributed to potentially modifiable risk factors. Cancers with the highest PAF, apart from cervical and Kaposi sarcoma, included larynx (85.6%), stomach (82.6%), liver (82.3%), lung (80.7%) and penis (75.0%). In contrast, cancers with lower PAF were ovarian cancer (8.4%), leukemia (12.8%), pancreatic cancer (21.8%), skin melanoma (25.0%) and bladder cancer (27.9%) (Table 2).

**Table 2:** PAF of cancer according to topography and potentially modifiable risk factors

TOPOGRAPHY	PAF (%)	C.I. 95%	% TOTAL PAF
Mouth, pharynx (C00-C14)			
HPV Infection	8.1	6.5–9.8	
Current tobacco use	20.5	11.8–30.8	65.7
Alcohol use	36.1	23.2–48.2	
Low fruit and vegetable consumption	1.1	0.1–2.2	
Esophagus (C15)			
Current tobacco use	11	6.9–15.9	
Obesity	12.3	3.9–36.3	60.8
Sedentary behavior	21.9	2.2–37.5	
Alcohol use	15.6	13.8–17.6	
Stomach (C16)			
*Helicobacter pylori* infection	46.4	18.6–65.9	
Current tobacco use	8.3	3.8–14.4	
Obesity	2.8	0.7–5.1	82.6
Red meat consumption	18.9	10.2–18.2	
Processed meat consumption	6.2	1.0–11.5	
Colorectal (C18-C21)			
Current tobacco use	2.1	1.1–3.1	
Sedentary behavior	18.5	8.3–27.1	
Alcohol use	4.1	2.1–6.3	
Obesity	4.1	2.4–6.1	53.6
Overweight	7.4	0.1–15.1	
Red meat consumption	5.9	1.5–9.8	
Processed meat consumption as	5	2.4–7.8	
Low fruit and vegetable consumption	6.6	0.9–12.4	
Liver (C22)			
Hepatitis B virus	32.4	30.2–34.6	
Hepatitis C virus	21.5	20.1–23.1	82.3
Current tobacco use	5.7	3.8–6.6	
Obesity	16.6	10.2–23.3	
Overweight	6	0.8–11.4	
Gallbladder (C23)			
Obesity	12.6	5.3–20.8	18.6
Overweight	6	2.6–9.6	
Pancreas (C25)			
Current tobacco use	7.3	6.1–8.4	
Alcohol use	7.2	4.7–9.6	21.8
Obesity	7.2	3.1–11.7	
Larynx (C32)			
HPV infection	22.1	10.9–37.0	85.6
Current tobacco use	38.8	18.5–60.6	
Low fruit and vegetable consumption	24.7	14.4–33.2	
Lung (C34)			
Current tobacco use	44.3	17.5–70.0	
Secondhand smoke	7.7	5.0–10.2	80.7
Sedentary behavior	17	4.4–28.3	
Low fruit and vegetable consumption	11.7	4.3–19.3	
Skin melanoma (C43)			
Ultraviolet radiation	25	20.7–29.4	25
Kaposi sarcoma (C46)			
HHV-8 infection	100	96.4–100.0	100
Breast (C50)			
Current tobacco use	9.2	1.2–25.8	
Secondhand smoke	5.6	2.1–9.0	
Obesity	8.5	6.3–10.8	41.8
Overweight	6	3.6–8.5	
Sedentary behavior	12.5	2.5–21.7	
Cervix (C53)			
HPV infection	100	96.4–100.0	100
Endometrium (C54.1)			
Obesity	28.4	22.3–34.7	58
Overweight	10.6	5.6–15.6	
Sedentary behavior	19	6.3–30 − 8	
Ovary (C56)			
Obesity	5.9	3.5–8.4	8.4
Overweight	2.5	5.6–13.2	
Penis (C60)			
HPV infection	75,0	45,6–88,5	75,0
Kidney (C64)			
Current tobacco use	4.6	2.7–6.5	36.2
Obesity	16.9	13.3–20.4	
Overweight	14.8	11.4–18.0	
Bladder (C67)			
Current tobacco use	20.8	18.0–23.6	27.9
Obesity	16.9	13.3–20.4	
Overweight	14.8	11.4–18.0	
Hodgkin’s Lymphoma (C81)			
Epstein Barr virus	59.7	34.1–79.1	63.8
HIV	0.7	0.2–1.9	
Current tobacco use	3.4	1.3–5.7	
Non-Hodgkin’s Lymphoma (C82-C85, C96.3)			
Epstein Barr virus	68	39.1–84.5	69.6
HIV	1	0.7–1.39	
hepatitis C virus	0.6	0.4–0.9	
Leukemias (C91-C95)			
Current tobacco use	4.1	2.3–6.0	12.8
Obesity	5.5	3.7–7.6	
Overweight	3.3	1.5–5.0	

The estimated fraction of cancer cases attributable to potentially modifiable risk factors was 34.5% in men and 42% in women, potentially preventing 25,308 cases of cancer (10,439 in men and 14,869 in women). In men, cancers with the highest PAF were Kaposi sarcoma, larynx (98.5%), lung (97.8%), liver (95.7%), mouth/pharynx (87.8%) and stomach (75.8%). In women, cancers with the highest PAF were cervix, Kaposi sarcoma, liver (73.7%), non-Hodgkin’s lymphoma (69.2%), stomach (66.3%), larynx (65.5%) and Hodgkin’s lymphoma (61.4%) (Table 3). The cancers with the highest number of preventable cases in men were stomach (2,526 cases), colorectal (1632 cases), lung (1,256 cases), non-Hodgkin’s lymphoma (1,134 cases) and liver (964 cases) and in women were cervix (4,270 cases), breast (2,868 cases), stomach (1,968 cases), colorectal (1,296 cases) and non-Hodgkin’s lymphoma (1,050 cases) (Table 3).

**Table 3:** PAF, cancer cases and deaths attributable to potentially modifiable risk factors in Peruvian men and women

TOPOGRAPHY	MEN				WOMEN			
	PAF (%)	C.I. 95%	Cases	Deaths	PAF (%)	C.I. 95%	Cases	Deaths
Mouth, pharynx	87.8	64.7–100.0	464	178	29.2	15.7–45.1	155	58
Esophagus	69.1	33.6–100.0	186	174	40.2	9.3–83.6	32	29
Stomach	75.8	33.6–100.0	2,526	2,018	66.3	25.8–100.0	1,968	1,532
Colorectal	72.5	32.2–100.0	1,632	845	54.3	23.0–81.8	1,296	652
Liver	95.7	75.6–100.0	964	967	73.7	57.0–91.4	797	787
Gallbladder	8.1	3.1–13.7	20	13	25.6	9.8–42.2	199	116
Pancreas	26.7	16.8–37.6	191	184	19.4	13.4–25.1	173	165
Larynx	98.5	53.0–100.0	165	95	65.5	32.9–100.0	43	24
Lung	97.8	46.7–100.0	1,388	1,256	41.6	13.2–100.0	611	542
Skin melanoma	46.9	43.8–50.0	301	97	1.2	0.9–2.7	8	2
Kaposi Sarcoma	100.0	96.4–100.0	297	56	100.0	96.4–100.0	53	19
Breast	NA	NA	NA	NA	41.8	15.6–75.8	2,868	762
Cervix	NA	NA	NA	NA	100.0	96.4–100.0	4,270	2,288
Endometrium	NA	NA	NA	NA	58.0	34.1–81.1	725	180
Ovary	NA	NA	NA	NA	8.4	9.0–21.6	106	66
Penis	75.0	45.6–88.5	214	60	NA	NA	NA	NA
Kidney	33.4	23.3–44.0	394	167	34.3	27.3–41.5	265	102
Bladder	36.7	28.9–44.7	267	89	15.1	9.1–21.2	57	23
Hodgkin’s Lymphoma	66.4	36.8–91.4	127	52	61.4	35.0–82.6	107	34
Non-Hodgkin’s Lymphoma	70.2	40.5–87.7	1,134	568	69.2	39.9–86.4	1,050	437
Leukemias	15.8	6.4–26.9	169	132	10.2	1.9–14.8	85	68
**TOTAL**			**10,439**	**6,951**			**14,868**	**7,886**

NA: Not applicable

Oncogenic infections constitute the primary group of modifiable attributable factors; potentially preventing up to 10,883 cancer cases annually (PAF: 16.6%). Controlling HPV infection could prevent up to 4,563 cases annually. The second most important group of modifiable factors in preventing cancer compromises other lifestyle factors (PAF: 14.2%), potentially preventing up to 9,362 cases annually; obesity is associated with the highest number of annual cancer cases, potentially preventing up to 2,087 cases if controlled. The third most important group of modifiable factors for cancer in Peru is tobacco exposure (PAF: 7.2%), contributing to 4,754 annual cases. Avoiding direct tobacco consumption could potentially prevent treating 3,348 cases of cancer annually. Finally, exposure to ultraviolet radiation stands as the least attributable modifiable factor (PAF: 0.5%) accountable for 309 cancers annually (Tables 4 and 5).

In 2018, 43.4% of cancer deaths in Peru were attributed to potentially modifiable risk factors, with percentages of 43.4% in men and 43.4% in women, resulting in 14,839 deaths (6,953 in men and 7,886 in women). Preventable cancer deaths by controlling modifiable factors include gastric cancer (2,018), lung (1,256), liver (967), colorectal (845) and non-Hodgkin lymphoma (568) in men; while in women, preventable deaths include cervical cancer (2,288), gastric cancer (1,532), liver cancer (787), breast cancer (762) and lung cancer (542) (Table 3).

**Table 4:** Number of cancer cases and deaths attributable to potentially modifiable risk factors in Peru distributed by genders

FACTOR CLUSTERING	MODIFIABLE RISK FACTORS	MEN		WOMEN		BOTH GENDERS	
		CASES	DEATHS	CASES	DEATHS	CASES	DEATHS
Tobacco (Lifestyle)	Current tobacco use	2,157	1,572	1,191	608	3,348	2,180
	Secondhand smoke	106	92	1,300	206	1,406	298
Other unhealthy lifestyle factors (Lifestyle)	Sedentary behavior	778	494	1,270	723	2,048	1,217
	Overweight	420	301	1,221	409	1,641	710
	Obesity	868	630	1,219	768	2,087	1,398
	Red meat consumption	761	573	702	507	1,463	1,080
	Processed meat consumption	319	226	304	204	623	430
	Low fruit and vegetable consumption	486	320	145	125	631	445
	Alcohol use	593	325	276	98	869	423
UV Radiation (Environment-lifestyle).	UV Radiation	301	97	8	2	309	99
Infections (Lifestyle)	*Helicobacter pylori*	1,213	971	1,464	902	2,677	1,873
	HBV	376	369	233	302	609	671
	HCV	227	218	67	234	294	452
	HPV	293	98	4,270	2,311	4,563	2,409
	HHV8	297	56	53	19	350	75
	HIV	29	14	11	5	40	19
	EBV	1,215	597	1,135	463	2,350	1,060
	**TOTAL**	**10,439**	**6,953**	**14,869**	**7,886**	**25,308**	**14,839**

In the present study, oncogenic infections are the main group of modifiable factors that determine cancer mortality in Peru (PAF: 19.2%) responsible for 6,559 deaths. Controlling HPV infection, could potentially prevent 2,409 deaths annually. The second group of modifiable factors comprises other unhealthy lifestyle factors (PAF: 16.7%) associated with 5,703 deaths annually. Addressing obesity could prevent 1,398 cancer deaths. The third factor impacting cancer mortality in Peru in 2018 was tobacco exposure (PAF: 7.2%), contributing to 2,478 deaths annually, with direct tobacco consumption associated to 2,180 cancer deaths annually. Lastly, exposure to ultraviolet radiation had a PAF of 0.3% and was responsible for 99 deaths (Tables 4 and 6).

**Table 5:** Fraction of cancer cases attributable to potentially modifiable risk factors in Peru distributed by gender

FACTOR CLUSTERING	MODIFIABLE RISK FACTORS	MEN		WOMEN		BOTH GENDERS	
		PAF CASES	PAF CLUSTERED	PAF CASES	PAF CLUSTERED	PAF CASES	PAF CLUSTERED
Tobacco (Lifestyle)	Current tobacco use	7.1	7.5	3.4	7.0	5.1	7.2
	Secondhand smoke	0.3		3.7		2.1	
Other unhealthy lifestyle factors (Lifestyle)	Sedentary behavior	2.6	13.9	3.6	14.5	3.1	14.2
	Overweight	1.4		3.4		2.5	
	Obesity	2.9		3.4		3.2	
	Red meat consumption	2.5		2.0		2.2	
	Processed meat consumption	1.1		0.9		0.9	
	Low fruit and vegetable consumption	1.6		0.4		1.0	
	Alcohol use	2.0		0.8		1.3	
UV Radiation (Environment-lifestyle).	UV Radiation	1.0	1.0	0.0	0.0	0.5	0.5
Infections (Lifestyle)	*Helicobacter pylori*	4.0	12.0	4.1	20.4	4.1	16.6
	HBV	1.2		0.7		0.9	
	HCV	0.7		0.2		0.4	
	HPV	1.0		12.1		6.9	
	HHV8	1.0		0.1		0.5	
	HIV	0.1		0.0		0.1	
	EBV	4.0		3.2		3.6	
**TOTAL**	**All risk factors**	**34.5**	**34.4**	**42.0**	**41.9**	**38.5**	**38.5**

**Table 6:** Fraction of cancer deaths attributable to potentially modifiable risk factors in Peru distributed by gender

FACTOR CLUSTERING	MODIFIABLE RISK FACTORS	MEN		WOMEN		BOTH GENDERS	
		PAF DEATHS	PAF CLUSTERED	PAF DEATHS	PAF CLUSTERED	PAF DEATHS	PAF CLUSTERED
Tobacco (Lifestyle)	Current tobacco use	9.8	10.4	3.3	4.5	6.4	7.2
	Secondhand smoke	0.6		1.1		0.9	
Other unhealthy lifestyle factors (Lifestyle)	Sedentary behavior	3.0	17.9	4.0	15.6	3.6	16.7
	Overweight	1.9		2.2		2.1	
	Obesity	3.9		4.2		4.1	
	Red meat consumption	3.6		2.8		3.2	
	Processed meat consumption	1.4		1.1		1.3	
	Low fruit and vegetable consumption	2.0		0.7		1.3	
	Alcohol use	2.0		0.5		1.2	
UV Radiation (Environment-lifestyle).	UV Radiation	0.6	0.6	0.0	0.0	0.3	0.3
Infections (Lifestyle)	*Helicobacter pylori*	6.1	14.5	5.0	23.3	5.5	19.2
	HBV	2.3		1.7		2.0	
	HCV	1.4		1.3		1.3	
	HPV	0.6		12.7		7.0	
	HHV8	0.3		0.1		0.2	
	HIV	0.1		0.0		0.1	
	EBV	3.7		2.5		3.1	
**TOTAL**	**All risk factors**	**43.4**	**43.4**	**43.4**	**43.4**	**43.4**	**43.4**

## Discussion

This study, conducted in 2018, prior to the COVID-19 pandemic in the population of 15 years old and older, revealed that 38.5% of new cancer cases and 43.4% of cancer-related deaths in Peru were attributable to potentially modifiable risk factors. Oncogenic infections, alongside unhealthy lifestyles, accounted for one-third of cancer cases and nearly two-fifths of cancer deaths. Notably, HPV infection, current tobacco use and *helicobacter pylori* infection emerged as the primary risk factors contributing to a higher number that cause greater number of cancer cases and deaths.

The fraction of cancer cases attributable to potentially modifiable risk factors in Peru lies between intermediate ranges compared to other countries. It is higher than countries such as Australia [28] (32.0%), Eastern Mediterranean countries [15] (33.4%), Japan [14] (35,9%) and Canada [29] (33–37%). However, it is lower than the US [9] (42.0%) and similar to the United Kingdom [12] (37.7%). Regarding cancer mortality, Peru’s fraction attributable to modifiable factors is intermediate compared to Japan [14], (41%) and lower than the US [10] (45.1%) and China [30] (45.2%). Brazil [16] stands out as the only country of Latin America with a PAF including oncogenic infections and unhealthy lifestyles among cancer risk factors. In terms of fraction attributable to cases, Peru exhibited a higher PAF compared to Brazil (38.4% versus 34.2%), while demonstrating a similar PAF for attributable deaths (43.4% Peru versus 42% in Brazil).

Oncogenic infections accounted for the highest PAF in Peru, contributing to 16.6% of cancer cases and 19.2% of deaths. This PAF is notably higher than those reported in other countries such as the USA [10], Canada [29], Australia [28] and the UK [12], where infections explain 3.3%, 3.7%, 2.9% and 3.6% of cancer cases, respectively. In Japan [14], the reported fraction is slightly lower at 16.6%. In Latin America, the study performed in Brazil [16] places oncogenic infections in second place, following tobacco, however, the exact PAF value wasn´t provided. The burden of modifiable risk factors for cancer in Peru is attributed to the “double burden of disease”, a common phenomenon in low- to middle-income countries facing many disparities. These countries must confront the increasing burden of non-communicable diseases, and unresolved communicable diseases rooted in structural health determinants [31–34].

This study underscores Peru’s struggle to control communicable diseases as a contributing factor to cancer despite existing policies, plans and strategies aimed at prevention, vaccination, screening and early detection of many associated neoplasms [1, 2].

The second highest PAF in Peru was attributed to unhealthy lifestyle (excluding tobacco consumption), similar to findings obtained by Islami in the US [9] for cancer cases (14.2% Peru versus 13.9% US) and slightly higher for cancer deaths (16.7% versus 14.9%). When including tobacco as a part of unhealthy lifestyle factors, Peru’s PAF was 21.4%, slightly lower than that reported by neighboring countries like Chile (30%) and Brazil (26.5%). These disparities found in the Region are attributed to the various levels of epidemiological transition among countries. Typically, countries with higher incomes and aging population tend to have a greater prevalence of unhealthy lifestyles [1, 5, 6, 35]. Currently, Chile boasts the highest aging rate in the Andean region, reaching 58%, placing it at stage 9 of transition according to the categorization proposed by CEPAL [36]. Meanwhile, Peru stands at stage 6 in this framework.

In Peru, the fraction attributable to modifiable factors such as tobacco and ultraviolet radiation for the occurrence of cancer cases and deaths is notably lower compared to most studies reported in other countries [10, 12, 14, 28, 29], including neighboring countries like Chile [23] and Brazil [16]. These findings are explained by successful tobacco control policies that have reduced consumption trends over the past two decades. However, poor access to diagnosis and under-reporting of lung cancer is possible, especially in rural remote regions away from the capital [2, 37]. On the other hand, UV radiation holds minimal relevance in Peru, as most skin melanomas are unrelated to UV radiation (acral melanomas) [38].

Our data underscores the imperative need to strengthen cancer prevention and control policies in Peru, emphasizing interventions proven to offer high cost/effectiveness. Prioritizing optimal vaccination coverage against oncogenic infections such as HPV and HBV [39, 40]. Simultaneously, implementing effective strategies to promote healthy lifestyles in the population, including reducing tobacco consumption, increasing physical activity, and fostering healthy dietary habits is essential [8, 37]. On the other hand, evidence from various researches, such as the analysis of mortality trends related to gastric cancer, demonstrates that improving access to safe water can significantly reduce the burden of gastric cancer attributed to *Helicobacter pylori* [34, 40–43].

This research significantly contributes to understanding cancer causality within the socio-health context, serving as valuable input for researchers, academics and decision-makers involved in the formulation of policies, plans, strategies and cost-effective interventions. We reaffirm the importance of adopting a territorial approach to address the most vulnerable regions, aiming to reduce cancer incidence and mortality. This approach optimizes the collective efforts of both the State and society.

One limitation of the PAF research model was the bias from the use of secondary information sources, that could result in some level of underreporting. To mitigate this, we relied on estimates of cases and deaths from the IARC, based on data from population-based cancer registries in Peru, which could have been affected in a smaller extent by the underreporting compared to vital records for deaths.

It should also be taken into account that PAF studies consider the effect of each risk’s factor in an independent way; it means, they do not consider possible interactions or confounding effects between factors, which constitutes a limitation of this methodology.

Another limitation was the scarcity of studies that reported the PAF for modifiable risk factors specifically for cancer deaths, published in the past decade. Most studies focused on PAF for new cancer cases, affecting the comparability of our results. However, leveraging previous studies conducted in Latin America and other regions allowed contextualization of the outcomes obtained for Peru.

There are some differences in the considered risk factors, compared to studies such as those by Islami (10 for the USA or Poirier [28] for Canada, which do not include Epstein Barr virus infection as a risk factor for Hodgkin’s and non-Hodgkin lymphoma, unlike this study that did include EBV. Additionally, prostate cancer was not included in our study due to insufficient evidence of modifiable risk factors. The fraction attributable to ionizing radiation could not be obtained due to a lack of exposed population data in Peru. Lastly, although firewood is used as cooking fuel in a fifth of Peruvian households, this study did not include exposure to firewood as a risk factor for lung cancer, as most PAF studies for this cancer did not consider it.

Interpreting the results obtained in this study requires caution, considering the existing methodological particularities between studies, which might not have been conducted within the same timeframe, age groups or included the same cancer locations.

## Conclusion

Oncogenic infections accounted for the highest PAF, contributing to 16.6% of cancer cases and 19.1% of deaths. The burden of modifiable risk factors for cancer in Peru is attributed to the “double burden of disease”. Despite existing policies, plans and strategies for prevention, vaccination, screening, and early detection of various related neoplasms, this study underscores the lack of control of communicable diseases as a cause of cancer in Peru.

In 2018, prior to the COVID-19 pandemic, 38.5% of new cases and 43.4% of cancer deaths in Peru were attributable to potentially modifiable risk factors in the population of 15 years old and older. Oncogenic infections, combined with unhealthy lifestyle choices, accounted for one-third of cancer cases and almost two-fifths of cancer deaths. HPV infection, current tobacco use, and *Helicobacter pylori* infection were identified as the primary risk factors contributing to a higher number of cancer cases and deaths.

## Implications of all the available evidence

Our findings underscore the urgency to strengthen cancer prevention and control policies in Peru. This involves prioritizing cost-effective interventions, such as achieving optimal vaccination coverage against oncogenic infections such as HPV and HBV. Simultaneously, implementing effective strategies to promote healthy lifestyles in the population, including reducing tobacco consumption, increasing physical activity, and fostering healthy dietary habits is essential. On the other hand, the strategy of improving access to safe water can significantly reduce the burden of stomach cancer attributable to *helicobacter pylori.*

## Data Availability

The datasets used and/or analysed during the current study available from the corresponding author on reasonable request.

## References

[1] Piñeros M, Ramos W, Antoni S, Abriata G, Medina LE, Miranda JJ (2017). Cancer patterns, trends, and transitions in Peru: a regional perspective. Lancet Oncol.

[2] Ramos W, Guerrero N, Medina J, Guerrero P. Análisis de la situación del Cáncer en el Perú, 2018. Lima: Ministerio de Salud del Perú; 2022. https://www.dge.gob.pe/epipublic/uploads/asis/asis_2020_27_120833.pdf

[3] International Agency for Reasearch on Cancer. Cancer Today. Lyon, IARC; 2018. https://gco.iarc.fr/today/home. Accessed 20 Dec 2013.

[4] Centro Nacional de Epidemiología, Prevención y Control de Enfermedades, Ministerio de Salud del Perú. Carga de Enfermedad en el Perú. Estimación de los años de vida saludable perdidos 2016. Lima: Ministerio de Salud del Perú. 2019. https://www.dge.gob.pe/portal/docs/tools/Cargaenfermedad2016.pdf. Accessed 20 Dec 2013.

[5] Valdez W, Miranda J, Ramos W (2011). State of the epidemiologic transition in Peru during 1990 and 2006. Rev Peru Epidemiol.

[6] Ramos W, Venegas D, Honorio H, Pesantes J, Arrasco J, Yagui M (2014). Non-communicable diseases: effect of major transitions and social determinants. Rev Peru Epidemiol.

[7] Centro Nacional de Epidemiología (2023). Prevención Y Control De Enfermedades, Ministerio De Salud Del Perú. Análisis De Situación De Salud Del Perú, 2021.

[8] De La Cruz-Vargas JA, Ramos W, Chanduví W, Espinoza R, Guerrero N, Loayza-Castro J (2020). Feasibility study to evaluate the proportion of cancer attributable to modificable risk factors in Peru and Latin America. Rev Fac Med Hum.

[9] Teh HS, Woon YL (2021). Burden of cancers attributable to modifiable risk factors in Malaysia. BMC Public Health.

[10] Islami F, Goding Sauer A, Miller KD, Siegel RL, Fedewa SA, Jacobs EJ (2018). Proportion and number of cancer cases and deaths attributable to potentially modifiable risk factors in the United States. CA Cancer J Clin.

[11] Brenner DR, Friedenreich CM, Ruan Y, Poirier AE, Walter SD, King WD (2019). The burden of cancer attributable to modifiable risk factors in Canada: methods overview. Prev Med.

[12] Brown KF, Rumgay H, Dunlop C, Ryan M, Quartly F, Cox A (2018). The fraction of cancer attributable to modifiable risk factors in England, Wales, Scotland, Northern Ireland, and the United Kingdom in 2015. Br J Cancer.

[13] Islami F, Chen W, Yu XQ, Lortet-Tieulent J, Zheng R, Flanders WD (2017). Cancer deaths and cases attributable to lifestyle factors and infections in China, 2013. Ann Oncol.

[14] Inoue M, Hirabayashi M, Abe SK, Katanoda K, Sawada N, Lin Y (2022). Burden of cancer attributable to modifiable factors in Japan in 2015. Glob Health Med.

[15] Kulhánová I, Znaor A, Shield KD, Arnold M, Vignat J, Charafeddine M (2020). Proportion of cancers attributable to major lifestyle and environmental risk factors in the Eastern Mediterranean region. Int J Cancer.

[16] Azevedo e Silva G, de Moura L, Curado MP, Gomes FS, Otero U, Rezende LFM (2016). The fraction of Cancer Attributable to Ways of Life, infections, Occupation, and Environmental Agents in Brazil in 2020. PLoS ONE.

[17] Pichon-Riviere A, Bardach A, Rodríguez Cairoli F, Casarini A, Espinola N, Perelli L, et al. Health, economic and social burden of tobacco in Latin America and the expected gains of fully implementing taxes, plain packaging, advertising bans and smoke-free environments control measures: a modelling study. Tob Control. 2023:tc–2022–057618. 10.1136/tc-2022-057618 10.1136/tc-2022-057618 PMC11503199 37142423

[18] Torres-Roman JS, Valcarcel B, Martinez-Herrera JF, Bazalar-Palacios J, La Vecchia C, Raez LE (2022). Mortality trends for Lung Cancer and Smoking Prevalence in Peru. Asian Pac J cancer Prevention: APJCP.

[19] Torres-Roman JS, Julca-Marín D, Ticona-Tiña D, Quispe-Vicuña C, Bazalar-Palacios J, De La Cruz-Ku G (2023). Trends in gastric cancer mortality 2005–2020 in Peru and its geographical areas: a joinpoint regression analysis. Cancer Epidemiol.

[20] Torres-Roman JS, De la Cruz-Ku G, Juárez-Leon V, Calderón-Solano D, Bazalar-Palacios J, Vecchia CL (2023). Mortality trends and geographic distribution of kidney cancer in Peru: a secondary analysis. BMC Urol.

[21] Torres-Roman JS, Ronceros-Cardenas L, Valcarcel B, Arce-Huamani MA, Bazalar-Palacios J, Ybaseta-Medina J (2021). Cervical cancer mortality in Peru: regional trend analysis from 2008–2017. BMC Public Health.

[22] Torres-Roman JS, Gomez-Rubio V, Sanchez-Trujillo L, Delgado-Rosas E, Puche-Vergara F, Sanz-Anquela JM (2020). Geographic study of mortality due to mesothelioma in Peru and its evolution. Cancer Epidemiol.

[23] Rezende LFM, Murata E, Giannichi B, Tomita LY, Wagner GA, Sanchez ZM (2020). Cancer cases and deaths attributable to lifestyle risk factors in Chile. BMC Cancer.

[24] GBD 2019 Cancer Risk Factors Collaborators (2022). The global burden of cancer attributable to risk factors, 2010-19: a systematic analysis for the global burden of Disease Study 2019. Lancet.

[25] Parkin DM (2011). The fraction of cancer attributable to lifestyle and environmental factors in the UK in 2010. Br J Cancer.

[26] Shin A, Park S, Shin HR, Park EH, Park SK, Oh JK (2011). Population attributable fraction of infection-related cancers in Korea. Ann Oncol.

[27] Arnold M, Lam F, Ervik M, Soerjomataram I. Cancers attributable to UV radiation. Lyon, France: International Agency for Research on Cancer; 2018. https://gco.iarc.fr/causes/uv

[28] Whiteman DC, Webb PM, Green AC, Neale RE, Fritschi L, Bain CJ (2015). Cancers in Australia in 2010 attributable to modifiable factors: summary and conclusions. Aust N Z J Public Health.

[29] Poirier AE, Ruan Y, Volesky KD, King WD, O’Sullivan DE, Gogna P (2019). The current and future burden of cancer attributable to modifiable risk factors in Canada: Summary of results. Prev Med.

[30] Feng RM, Zong YN, Cao SM, Xu RH (2019). Current cancer situation in China: good or bad news from the 2018 Global Cancer statistics?. Cancer Commun (Lond).

[31] Bygbjerg IC (2012). Double burden of noncommunicable and infectious diseases in developing countries. Science.

[32] Boutayeb A (2006). The double burden of communicable and non-communicable diseases in developing countries. Trans R Soc Trop Med Hyg.

[33] Thun MJ, DeLancey JO, Center MM, Jemal A, Ward EM (2010). The global burden of cancer: priorities for prevention. Carcinogenesis.

[34] Venegas-Ojeda D, Agüero-Palacios YD (2021). Gastric cancer mortality rate trend in Peru: segmented regression model from 1995 to 2013. Rev Fac Med Hum.

[35] Szot Meza J (2003). Demographic-epidemiologic transition in Chile, 1960–2001. Rev Esp Salud Publica.

[36] Turra C, Fernandes F. La transición demográfica: oportunidades y desafíos en la senda hacia el logro de los Objetivos de Desarrollo Sostenible en América Latina y el Caribe, Documentos de Proyectos (LC/TS.2020/105). Santiago, Comisión Económica para América Latina y el Caribe (CEPAL); 2021. https://www.cepal.org/es/publicaciones/46805-la-transicion-demografica-oportunidades-desafios-la-senda-logro-objetivos

[37] Torres-Roman JS, Valcarcel B, Martinez-Herrera JF, Bazalar-Palacios J, La Vecchia C, Raez LE (2022). Mortality trends for Lung Cancer and Smoking Prevalence in Peru. Asian Pac J Cancer Prev.

[38] Basurto-Lozada P, Molina-Aguilar C, Castaneda-Garcia C, Vázquez-Cruz ME, Garcia-Salinas OI, Álvarez-Cano A (2021). Acral lentiginous melanoma: Basic facts, biological characteristics and research perspectives of an understudied disease. Pigment Cell Melanoma Res.

[39] Palladini A, Landuzzi L, Lollini PL, Nanni P (2018). Cancer immunoprevention: from mice to early clinical trials. BMC Immunol.

[40] Lollini PL, Nicoletti G, Landuzzi L, Cavallo F, Forni G, De Giovanni C (2011). Vaccines and other immunological approaches for cancer immunoprevention. Curr Drug Targets.

[41] Castillo M, Bernabe L, Castaneda CA, Chavez I, Ruiz E, Barreda F (2019). *Helicobacter Pylori* detected in tap water of Peruvian patients with gastric Cancer. Asian Pac J Cancer Prev.

[42] Eichelberger L, Murphy G, Etemadi A, Abnet CC, Islami F, Shakeri R (2015). Risk of gastric cancer by water source: evidence from the Golestan case-control study. PLoS ONE.

[43] Klein PD, Graham DY, Gaillour A, Opekun AR, Smith EO (1991). Water source as risk factor for *Helicobacter pylori* infection in Peruvian children. Gastrointest Physiol Working Group Lancet.

